# Synonymous mutations that regulate translation speed might play a non-negligible role in liver cancer development

**DOI:** 10.1186/s12885-021-08131-w

**Published:** 2021-04-09

**Authors:** Qun Li, Jian Li, Chun-peng Yu, Shuai Chang, Ling-ling Xie, Song Wang

**Affiliations:** Department of interventional radiology, The Affiliated Hospital of Qingdao University, Qingdao, China

**Keywords:** Liver cancer, Synonymous mutations, Codon usage, Gene expression, Translation

## Abstract

**Background:**

Synonymous mutations do not change the protein sequences. Automatically, they have been regarded as neutral events and are ignored in the mutation-based cancer studies. However, synonymous mutations will change the codon optimality, resulting in altered translational velocity.

**Methods:**

We fully utilized the transcriptome and translatome of liver cancer and normal tissue from ten patients. We profiled the mutation spectrum and examined the effect of synonymous mutations on translational velocity.

**Results:**

Synonymous mutations that increase the codon optimality significantly enhanced the translational velocity, and were enriched in oncogenes. Meanwhile, synonymous mutations decreasing codon optimality slowed down translation, and were enriched in tumor suppressor genes. These synonymous mutations significantly contributed to the translational changes in tumor samples compared to normal samples.

**Conclusions:**

Synonymous mutations might play a role in liver cancer development by altering codon optimality and translational velocity. Synonymous mutations should no longer be ignored in the genome-wide studies.

## Key message

Synonymous mutations play a role in liver cancer development by altering codon optimality and translational velocity.

## Background

By definition, synonymous mutations are those mutations that do not change amino acids, and they usually take place at the third codon positions [1, 2]. Classic evolutionary theories only consider the functional consequence of missense mutations, and the synonymous mutations are regarded as neutral sites and are used as “control group” to test the selection force on missense mutations [3–5]. Based on this notion that only missense mutations could have functional impacts s (among point mutations, not including Indels or structural changes), researchers tend to automatically ignore the synonymous mutations and only put efforts in studying the protein-changing variations [6, 7]. The best example could be seen in the recent outbreak of COVID-19 pandemic, where many mutation-based functional studies only focused on missense mutations in the SARS-CoV-2 genome [8], while the synonymous mutations/codons in SARS-CoV-2 were much less noticed [9, 10].

Recently, molecular evidence showed that synonymous mutations actually have their functional impact. This idea challenged the classic evolutionary theory. As we know, synonymous codons are not equally used by the genome. An organism tends to favor a particular set of synonymous codons [11], and these favored codons usually have higher frequencies in the genome [12–14], termed optimal codons [15, 16]. Synonymous mutations could switch a rarely used codon to a frequently used codon, changing the codon optimality. If the optimal codons are advantageous, then the synonymous mutations would be subjected to natural selection as they alter the codon optimality [17]. In silico, in vitro, and in vivo evidence all showed that optimal codons have faster translational velocity compared to the non-optimal codons [18–23]. These striking findings indicated that although the synonymous codons encode the same amino acid, they differ in translational speed. In other words, proteins translated from different synonymous codons are qualitatively equal but quantitatively different.

Given the functional impact of synonymous mutations, there is no reason to exclude these mutations from the studies related to human diseases including cancer. There are already a bunch of studies reporting the disease-related synonymous mutations. These studies could be classified into the following categories based on their methodology and themes. (1) Bioinformatic analyses studying the global patterns of the collective effect of synonymous mutations. For example, genome-wide analysis revealed that a part of synonymous mutations could affect mRNA splicing and might contribute to cancer progression [24]. Evolutionary study indicated that synonymous mutations in oncogenes were also suppressed due to undetermined selection pressure [25]. The synonymous codon usage in cancers tends to avoid expensive and low-efficiency codons and prefers to use cheap and high-efficiency counterparts [26]. (2) Investigation or experimental verification of a particular or a few synonymous mutation(s) related to particular phenotype. For example, a single synonymous mutation observed in fragile X patient could alter the host gene expression and eventually lead to the syndrome [27], synonymous mutations in *PKD1* gene caused autosomal dominant polycystic disease [28], synonymous mutations in *ATP7B* gene was associated with Wilson disease [29], and somatic synonymous mutations contributed to melanoma [30, 31].

The above descriptions have connected the genotype with phenotype. At molecular level, the effects of synonymous mutations could be exerted via various ways [32] including (1) splicing changes [28, 29], (2) RNA structural changes [33–35], (3) altered translational speed [36], and (4) miRNA binding gain or loss [37]. Of note, in some systems or software the mutations related to splicing changes are listed separately although they belong to synonymous mutations [38].

The effect of synonymous mutations on translational speed is of our interest. More specifically, the mechanism underlying how synonymous mutations affect translational speed might be the alteration of tRNA concentration decoding the codon, which usually changes from tissue to tissue [39], differs between malignant and non-malignant cells [40], varies from species to species [41].

Based on our own field of liver cancer, there is urgent need for us to elucidate whether synonymous mutations play a role in liver cancer development. So far, the exon sequencing of patients only focused on a few missense variations in candidate genes. If synonymous mutations really contribute to liver cancer progression, then the existing exon sequencing methodology could omit a large number of causal mutations on synonymous sites. Obviously, the human genome has one third of synonymous mutations in coding regions, and therefore, the omission of synonymous mutations would be a great loss to the cancer field, and meanwhile misleading the diagnosis.

We aim to systematically verify the notion that synonymous mutations are not silent due to the ability to change codon optimality and translational speed. By using the tumor samples, where mutations are supposed to be more prevalent than randomly picked healthy populations, the observation of synonymous mutation-mediated translational changes would be of greater functional impact and biological significance. Moreover, the associations between the codon features (synonymous mutations, codon optimality, and translational speed) and the gene enrichment (oncogene or tumor suppressor gene) would increase the confidence that these synonymous mutations might play a non-negligible role in tumorigenesis.

In this article, we utilized the transcriptome (RNA-seq) and translatome (ribosome profiling) of liver cancer and normal tissue from ten patients (GSE112705) [42]. We found that synonymous mutations that increase the codon optimality could significantly enhance the translational velocity, and are enriched in oncogenes. The synonymous mutations that decrease codon optimality slow down translation, and are enriched in tumor suppressor genes.

## Methods

### Data collection

The human and macaque reference genomes were downloaded from Ensembl website (http://ensemblgenomes.org/). The sequencing data (RNA-seq and ribosome profiling) of normal/tumor tissues from ten patients were downloaded from NCBI via accession number GSE112705 [42]. According to the original literature, the ten patients suffered from hepatocellular carcinoma (HCC). The liver tumor tissues and their adjacent normal liver tissues were collected.

The lists of oncogenes and tumor suppressor genes (TSG) were downloaded from the cancer gene consortium website (CGC, https://cancer.sanger.ac.uk/census/). Totally 240 oncogenes and 242 TSG were obtained. This final gene list did not include the ambiguously annotated genes, for example, some genes were annotated as both oncogene and TSG. These genes were not included in our list.

The SNP data of the 1000 genome project were downloaded from official website (ftp://ftp.1000genomes.ebi.ac.uk/). The genome version is “phase2 reference assembly sequence hs37d5”.

### Mapping and variant calling

We mapped the sequencing reads to the human reference genome by using STAR [43] with default parameters (version 2.7.3a). The gene expression profile was directly downloaded from the original article [42]. Mutations were called by Samtools [44] with parameter -Q 30 -q 20 (version 1.10). The parameters require the base quality to be higher than 30 and mapping quality higher than 20. Higher base quality represents lower sequencing error probability. Q > 30 means error probability <1e-3. Mapping quality increases with the reliability of the sequence alignment. Mapping quality q > 20 almost ensures a read to be uniquely mapped to the reference genome.

As we have defined in the main text, the direction of mutations should be “from ancestral allele to derived allele”. To obtain the tumor-specific SNP, we required the mutations to have both the ancestral and derived alleles in tumor RNAs. Meanwhile, this position in normal sample should be covered by at least five reads (of ancestral allele) to prove that this site did not have mutation in normal tissues. This pipeline was first applied to the data of each of the ten patients to get the SNP profiles of ten individuals. Next, these ten SNP sets were merged to get a union of SNP sites, which is approximately 400,000 unique sites. Functional annotation was done by software SnpEff [38].

For the so-called tumor-specific mutations in this study, there are two layers. In the section that displayed the mutation landscape in RNA-seq, we only required (1) sequencing coverage ≥5 in normal tissues and no mutations were detected; (2) coverage ≥5 in tumor samples and mutations were detected. In the section that compared translational speed of different alleles (which is sensitive to low sequencing coverage), we further required the mutation sites to have (1) RNA-seq coverage ≥20 in normal tissues and no mutation was detected; (2) RPF (A-site) coverage ≥20 in normal tissues and no mutation was detected in A-site tri-nucleotide; (3) RNA-seq coverage ≥20 in tumors and both two alleles were detected with allele count ≥3; (4) RPF (A-site) coverage ≥20 in tumors and both two alleles were detected in A-site tri-nucleotide with allele count ≥3; (5) absent in 1000-genomes. We have emphasized that we do not intend to obtain an accurate list of tumor-specific mutations. Instead, we just want to identify the bona fide ones to perform translational analysis and prove the notion that synonymous mutations could affect translational speed.

### Determining P-site offset and A-site RPF density

With similar idea and methodology to previous studies [45, 46], we used the A-site tri-nucleotide to calculate the ribosome density and translation elongation speed of each codon. We obtained the RPF alignment file and ran with software plastid [47]. The outcome of this pipeline would tell us the P-site offset values for each length of the RPF reads. By trimming the offset from the 5′ end, one could determine the P-site tri-nucleotide in each RPF read. The A-site tri-nucleotide is just located downstream the P-site in each RPF read. For the ribosome density used to calculate translational speed and the mutations detected in RPF reads, only the A-site tri-nucleotide was used. Therefore, the RPF density on each codon was also termed A-site density in this article.

### Translation efficiency

The TE of genes was calculated as RPKM_RPF_/RPKM_mRNA_. RPKM stood for “reads per kilobase per million mapped reads”. The raw reads count of each gene was accomplished by software package htseq [48].

### Conservation analysis

For each mutation site in human, the orthologous nucleotides in macaque were transferred by liftOver chain downloaded from Ensembl. The liftOver tool allows the transfer of genomic coordinates of different species. Command “liftOver human_sites human_to_macaque_chain macaque_sites”. When the orthologous location in the macaque genome is known, then we could extract the corresponding nucleotide using Bedtools [49]. Command “bedtools getfasta -fi macaque.fa -bed macaque_sites -fo macaque_nucleotide”. The dN (nonsynonymous substitution rate) was calculated by software yn00 [50]. When running yn00, first we need to have the orthologous gene table between human and macaque. This could be obtained by transferring the genomic coordinates of the two species and retrieve the gene ID with the help of the genome annotation file (gft format).

### Graphic works and statistics

The graphic works and statistics were realized in the R environment (version 3.5.2). KS tests were realized with command “ks.test()”. Correlation tests were performed with command “cor.test()”.

## Results

### SNP profile of the samples

**Fig. 1:**
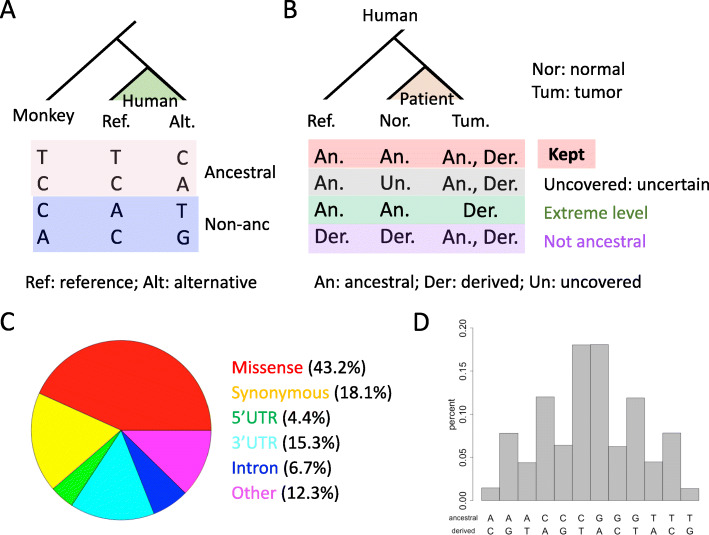
Definition and statistics of SNPs. **a** For SNPs in each sample, we require the human reference sequence to be identical to the sequence in macaque (*Macaca mulatta*) so that the alternative allele would be the derived allele. This is the rational way to confirm the direction of mutations. **b** To define the tumor-specific mutations, we require the normal samples to have only the ancestral allele and the tumor sample to have both ancestral and derived alleles. Sites with insufficient sequencing coverages in normal samples were uncertain and excluded (grey). Sites with 100% mutation levels in tumor samples were also unreliable and discarded (green). Non-ancestral sites (purple, very few) were not considered. **c** Fraction of SNPs regarding their functional annotation. **d** Percentage of SNPs regarding the nucleotide change of the mutation

By mapping the reads to the human reference genome and performing variant calling, we defined the mutations appearing in cancer tissues but absent in normal tissues (Fig. 1a, b). We stress that this study does not aim to retrieve the accurate full list of specific mutations in tumors. We only aim to find solid evidence that the synonymous mutations indeed affect the translational speed. To accomplish this, we wish to find the “high-confidence sites” where no derived mutations are observed in normal tissues while the tumor samples are heterozygous (Fig. 1b). Sites with low sequencing coverage are not informative to determine whether they are mutated or not, so we will perform stricter filtering criteria in the following sections when using ribosome profiling data, where the 1000-genomes SNPs are also excluded (see Methods). Here, we first show the landscape of mutations in RNA-seq.

The nucleotides in human reference genome might not reflect the ancestral state, and therefore we only keep the derived mutations by looking at the orthologous site in macaque (*Macaca mulatta*) (Fig. 1a). This step is necessary since the direction of mutations should not be based on the reference genome and could only be inferred from the sequence of outgroup species. In the comparison of normal and tumor tissues, we require the reference genome and normal tissue to be ancestral nucleotide and tumor tissue to have both the ancestral and derived nucleotides (Fig. 1b). The mutations in tumors with extreme high level of 100% were unreliable. Moreover, a SNP might be missed in normal tissues due to insufficient sequencing coverage. Therefore, when searching for these tumor tissue-specific mutations, we require them to have at least 5 RNA-seq reads covered in normal tissues (see Methods for details). Again, we re-emphasize that the so-called tumor-specific mutations might be overestimated due to insufficient sequencing coverage in normal tissues. However, we do not aim to acquire the accurate list of tumor-specific mutations. Instead, these mutations just serve as candidate sites for our downstream analysis on ribosome profiling data. In the following sections (where stricter cutoffs are used and the 1000-genomes SNPs are excluded. See Methods), we will observe that those synonymous sites are un-mutated in our normal tissues (coverage ≥20) but mutated in tumor samples with high confidence (coverage ≥20 & alternative allele ≥3), and that they indeed affect the translational speed by comparing different alleles. This is our ultimate purpose.

Here, under coverage ≥5 in RNA-seq of normal tissues and mutations detected in tumor sample, 9567 ~ 66,173 SNPs were found in the ten patients, with individual LC034 having the fewest SNPs and individual LC502 having the most SNPs. The union of these SNPs is 399,727 unique sites. Among these 399,727 unique SNPs in patients, 146,595 (43.2%) were missense, 61,259 (18.1%) were synonymous, ~ 20% were located in UTRs (untranslated regions), and ~ 20% were located in intron or other noncoding regions (Fig. 1c). The SNP profile show that transitions take place much more frequently than transversions (Fig. 1d), which confirms with our commonsense. These results represent the basic landscape of mutations detected in RNA-seq. The next step is to identify the signal of translation of these mutations, where stricter criteria and higher cutoffs will be used.

### Determining A-site tri-nucleotide in RPF reads

**Fig. 2:**
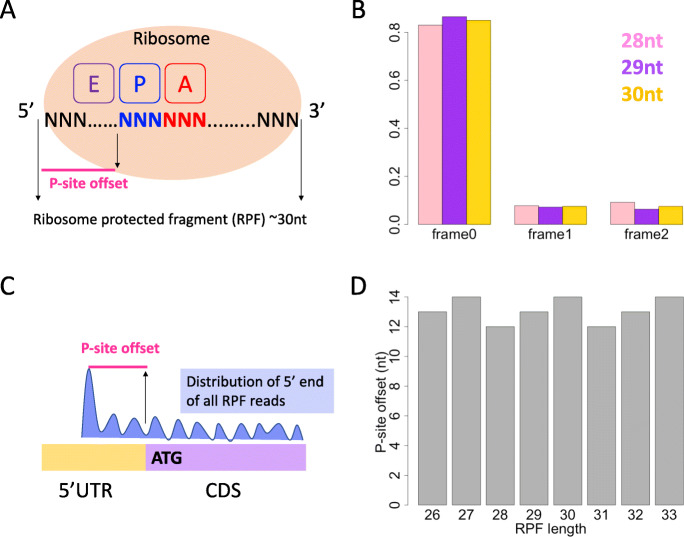
Illustration of what P-site and A-site mean and how offset is determined. **a** The ribosome protected fragment is usually around 30 nt. The positions being translated are P-site (3 nt) and A-site (3 nt). A-site should be used to calculate the translational speed of each codon. The distance from the 5′ end of the RPF to the 5′ of P-site is defined as “P-site offset”. When the position of P-site is determined, A-site is just 3 nt downstream of the P-site. **b** In the ribosome profiling data, we counted the percentages of RPF reads mapped to the different frames. Frame0 represents the RPF reads with 5′ end mapped to the first codon position. Frame1 means the 5′ end of RPF reads was mapped to the second codon position. Frame2 means the third codon position. **c** The 5′ end of RPF reads usually peaks at the upstream of start codon ATG. The distance from the peak to the start codon is the P-site offset. **d** The P-site offset of RPF reads with different lengths

The ribosome protected fragment, termed RPF, is typically around 30 nt. The position being translated is the P-site and A-site (Fig. 2a). P-site is more relevant to the amino acid property and A-site usually reflects the translational speed of the codon. The distance from the 5′ end of RPF read to the 5′ of P-site is termed P-site offset. Multiple tools [47, 51] are able to find out the P-site offset in the ribosome profiling data with good phase.

We looked at the phase of the ribosome profiling data in hand. For different RPF length, most of the reads have 5′ end mapped to the first codon position (Fig. 2b), suggesting the success of nucleotide digestion in the ribosome profiling protocol. Next, we employed software plastid [47] to determine the P-site offset RPF reads with different length (Fig. 2c, d). By trimming the offset from the 5′ end, we determine the P-site tri-nucleotide in each RPF read. The A-site tri-nucleotide is just located downstream the P-site in each RPF read (see Methods for details). In the following sections, only the A-site tri-nucleotide was used to calculate the ribosome density (translational speed) and the mutations detected in RPF reads.

### Capture of allele-specific translational events of synonymous mutations

**Fig. 3:**
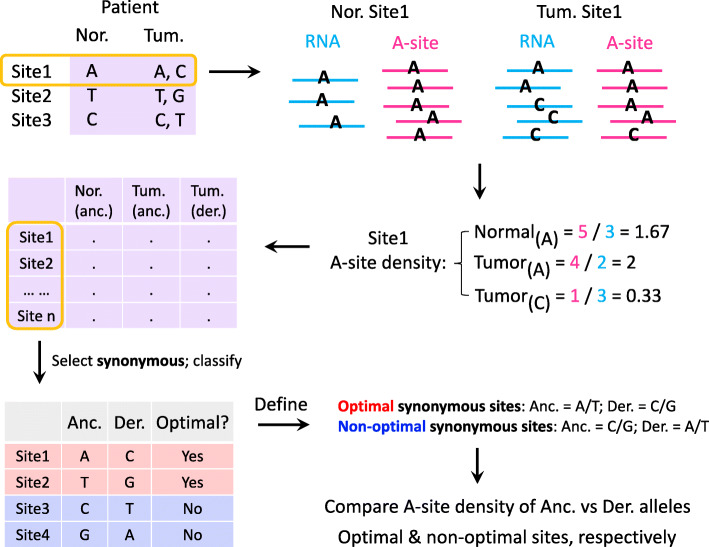
Illustrating the pipeline to compare the allele-specific RPF density (A-site tri-nucleotide density). According to the description in previous parts, the allele in normal tissue is the ancestral allele, and the tumor tissues have both the ancestral and derived allele. The A-site and RNA reads count could be extracted from the sequencing data. The A-site/RNA (A-site density) is calculated for each allele. We also classify the synonymous mutations based on codon optimality. Synonymous mutations from A/T to C/G are optimal and the opposite direction are non-optimal

For all the mutations we selected, the nucleotides in normal tissues are the ancestral alleles and the nucleotides in tumors have both ancestral and derived alleles (Fig. 3). For each of these alleles, it has RNA coverage and RPF (A-site) coverage, and the RPF (A-site) density could be calculated to represent translation elongation velocity (Fig. 3). Higher A-site density represents slower local translation velocity. In normal tissues, the A-site densities of the ancestral alleles are calculated, and in tumors, both the ancestral and derived alleles have their own RPF densities (Fig. 3). We calculated the A-site densities of the alleles for all SNP sites. Next, we retrieved the synonymous sites and check whether the synonymous mutations increase or decrease the codon optimality. Synonymous mutations from A/T to C/G increase codon optimality and those from C/G to A/T decrease codon optimality (Fig. 3). Our aim is to examine if the alteration in codon optimality could impact the A-site density, that is, the local translation elongation speed. As shown in Fig. 3, the comparison of A-site densities between normal and tumor samples might be questionable as we did not perform “normalization by library size”. However, the comparison of A-site densities between the two alleles in tumor sample is completely unbiased and valid because they are produced from the same sequencing library. Moreover, heterozygous SNPs within the same sample are exposed to the same trans environment, which provides the perfect condition to study the effects of cis elements like sequence features. Nevertheless, although the cross-sample comparison is affected by library size and other variables, we intend to add it as supporting evidence.

In the previous section that displayed the mutation landscape in RNA-seq, we only required sequencing coverage ≥5 and mutation detected in tumor samples. In the following comparison of translational speed, which would be sensitive to low sequencing coverage, we further required the mutation sites to have (1) RNA-seq coverage ≥20 in normal tissues and no mutation was detected; (2) A-site coverage ≥20 in normal tissues and no mutation was detected in A-site tri-nucleotide; (3) RNA-seq coverage ≥20 in tumors and both two alleles were detected with allele count ≥3; (4) A-site coverage ≥20 in tumors and both two alleles were detected in A-site tri-nucleotide with allele count ≥3; (5) absent in 1000-genome. We believe these criteria would ensure the high-confidence mutation sites we want. Now that we have defined the synonymous mutations in RNA-seq and RPF data, the next step would be the comparison of translational speed on the different alleles of the mutation sites.

### Optimal synonymous mutations increase local translational speed

**Fig. 4:**
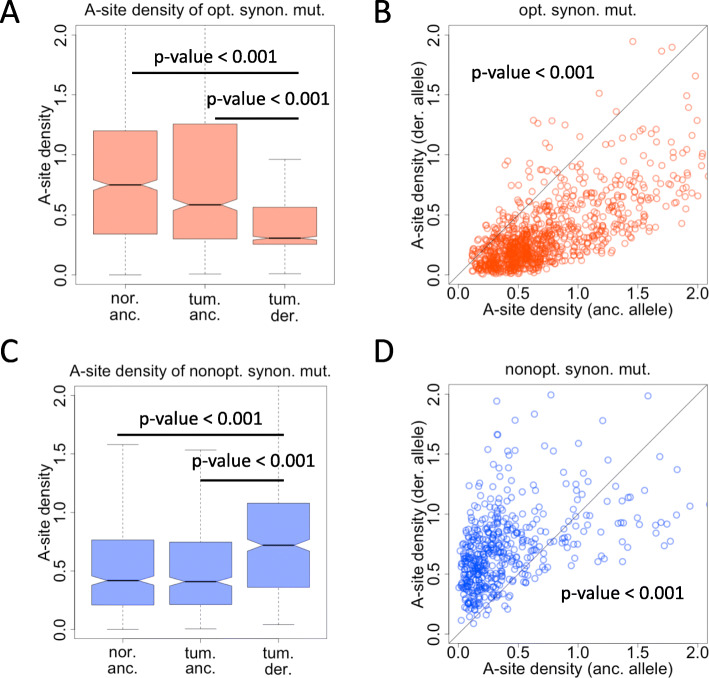
RPF density (A-site density) on different alleles of synonymous sites. **a** For optimal synonymous sites, the A-site densities on ancestral alleles in normal sample, ancestral alleles in tumor sample, and derived alleles in tumor sample were plotted. KS tests were used to calculate the *p*-values. **b** For optimal synonymous sites, the A-site densities on two alleles in tumor sample were compared. Paired t-test was used to calculate the p-value. **c** For non-optimal synonymous sites, the A-site densities on ancestral alleles in normal sample, ancestral alleles in tumor sample, and derived alleles in tumor sample were plotted. KS tests were used to calculate the p-values. **b** For non-optimal synonymous sites, the A-site densities on two alleles in tumor sample were compared. Paired t-test was used to calculate the p-value

We plotted the A-site density on each allele in normal and tumor samples. We first looked at the optimal synonymous mutations from A/T to G/C. Apparently, the A-site densities on ancestral alleles in normal and tumor samples are comparably high, while the A-site density on derived allele in tumors is lower (Fig. 4a). Since lower A-site density denotes faster translation elongation speeds, this result demonstrates that the optimal synonymous mutations really change the local translation rate. One may argue that the comparison between two samples did not consider the library size. Therefore, we display the A-site density of two alleles within the tumor samples (Fig. 4b). In the figure, each dot represents one optimal synonymous mutation site, and we could clearly see that the A-site density on ancestral allele (A/T) is significantly higher than that on derived allele (C/G). This is direct observation of the different translational speed on two alleles with different codon optimality (within the same library).

Similarly, we then looked at the non-optimal synonymous mutations from C/G to A/T. The A-site densities on ancestral alleles in normal and tumor samples are low, while the A-site density on derived allele in tumors is high (Fig. 4c). Again, to cancel the potential bias caused by library size, we compared the A-site densities on ancestral and derived alleles in tumors. The translation on ancestral alleles (C/G) is significantly faster than that on derived alleles (A/T) (Fig. 4d). This consolidates the notion that synonymous mutations in tumors altered the codon optimality and local translation elongation speed.

Note that the pre-filters on sequencing coverage (≥ 20) ensured the high confidence of these mutation sites where the normal tissues do not have mutations and the tumor samples have both alleles sequenced with adequate coverage (≥ 3). These criteria should make our results and conclusions more reliable.

### Enrichment of speed-controlling synonymous mutations in oncogenes and tumor suppressor genes

By merely observing the differential translation speed on two alleles in tumors does not prove the causal relationship between synonymous mutations and oncogenesis. However, the direct evidence could only be obtained from molecular and cellular experiments or animal models. Given the transcriptome and translatome data in hand, what we could do is to examine the enrichment of these synonymous mutations in different groups of genes. We denote the optimal (A/T to C/G) and non-optimal (C/G to A/T) synonymous mutations as the speed-controlling synonymous mutations (as proved by the results above).

The synonymous mutations used in this section was those with RNA-seq coverage ≥5. Although in the translational speed comparison we used a more stringent filter to obtain speed-affecting mutations with higher confidence, we believe that many of the discarded sites in the filtering steps would also affect translational speed and they are removed solely due to the technical issue (insufficient sequencing coverage). Therefore, in this section that compares the synonymous SNP density in different genes, to increase statistic power, we used the larger set of mutation sites with RNA-seq coverage ≥5. The results of high-confidence mutation sites will be displayed in the next section.

**Fig. 5:**
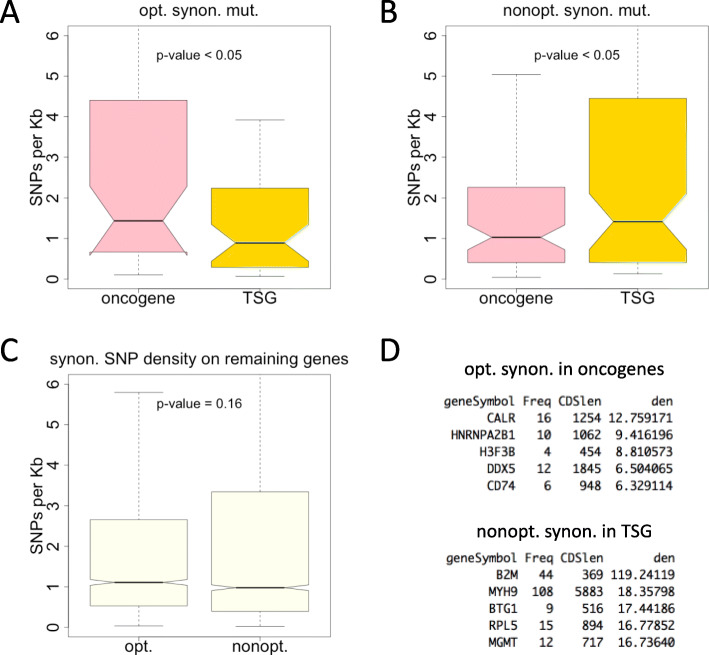
SNP density (number of SNPs per Kb CDS) in different genes. **a** Optimal synonymous SNP density in oncogenes and TSG. KS test was used to calculate p-value. **b** Non-optimal synonymous SNP density in oncogenes and TSG. KS test was used to calculate p-value. **c** Synonymous SNP density in the remaining genes. KS test was used to calculate p-value. **d** Examples of genes with high synonymous SNP density. Oncogenes with the highest optimal synonymous SNP density were shown. TSG with the highest non-optimal synonymous SNP density were shown. “Freq” means number of SNPs

We defined SNP density as the number of SNPs per Kb of CDS. Here, SNP means the tumor-specific SNPs as defined previously (and throughout this study). Meanwhile, we retrieved the lists of oncogenes and tumor suppressor genes (TSG) from cancer gene consortium (CGC, https://cancer.sanger.ac.uk/census/). Totally 240 oncogenes and 242 TSG were obtained. We found that the density of optimal synonymous mutations is significantly higher in oncogenes than in TSG (Fig. 5a, pooled samples are shown), and the density of non-optimal synonymous mutations is significantly higher in TSG than in oncogenes (Fig. 5b). Apart from oncogenes and TSG, the remaining genes also have these synonymous mutations. However, we did not see differential SNP densities (optimal versus non-optimal) on these genes (Fig. 5c).

These results make sense and seem to connect synonymous mutations to liver cancer development. In tumor samples, optimal synonymous mutations increase translational speed and are enriched in oncogenes, and non-optimal synonymous mutations decrease translational speed and are enriched in TSG, while other genes do not exhibit different densities of these optimal or non-optimal synonymous mutations.

To visualize our results and provide more specific examples, we listed the genes with the highest synonymous SNP density (Fig. 5d). Oncogenes with the highest density of optimal synonymous mutations and TSG with the highest density of non-optimal synonymous mutations were listed. Among the top genes, the oncogenes *CALR* (Calreticulin), *HNRNPA2B1* (Heterogeneous Nuclear Ribonucleoprotein A2/B1), and TSG including *B2M* (Beta-2-Microglobulin), *BTG1* (B-Cell Translocation Gene 1) may all play crucial roles in maintaining or disrupting the equilibrium status in normal cells. For example, mutations in gene *CALR* were reported to cause breast and colorectal cancer [52], ovarian carcinoma [53], and prostate cancer [54], and the mutations reported were missense mutations. Now that we found many optimal synonymous mutations in liver cancer tissues, this might broaden the knowledge about this gene and serve as guidance in diagnosis in the future.

**Fig. 6:**
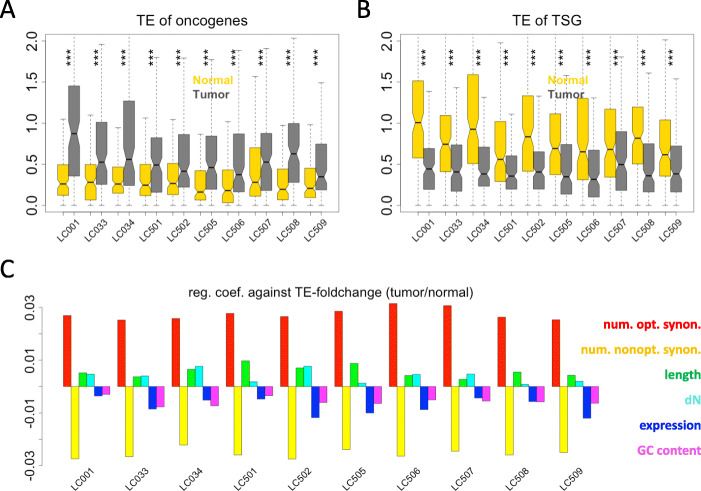
Translation efficiency (TE) in normal and tumor samples. **a** TE of oncogenes in normal and tumor samples. “***” represented p-value < 0.001. **b** TE of TSG in normal and tumor samples. **c** Regression coefficients of “Y ~ X1 + X2 + ...Xn”. Y is the TE fold change of tumor samples to normal samples. The variables were scaled to the same range of − 1 to 1. The variables included number of optimal synonymous mutations, number of non-optimal synonymous mutations, gene length, conservation level (dN), gene expression, and GC content

To show the potential effect of the synonymous mutations on the global translation efficiency (TE) of genes, we first compared the TE in tumor samples and normal samples. For oncogenes, the TE was significantly higher in tumor samples than normal samples (Fig. 6a). For TSG, the TE was significantly higher in normal samples than tumor samples (Fig. 6b). Since we already observed the enrichment of “speed-controlling” synonymous mutations in these gene sets, it was reasonable to attribute the TE foldchange to the effect of synonymous mutations. We performed multiple regression analysis “Y ~ X1 + X2 + ... + Xn”, where Y was the TE foldchange between tumor and normal samples, and X1 to Xn were the variables. We aimed to see which variable had the strongest effect on TE foldchange. We found that the number of optimal synonymous mutations positively contributed to TE foldchange and that the number of non-optimal synonymous mutations negatively contributed to TE foldchange (Fig. 6c). For other variables like gene length, conservation level (dN), expression, and GC content played minor roles in determining the tumor versus normal translational changes. This observation strongly indicated that synonymous mutations did have impact on the local and global translational changes between tumor and normal samples.

### Robust patterns are found for the high-confidence mutations in tumors

As stated, the so-called tumor-specific mutations have two layers in this article. The results of the mutations with loose criteria (coverage ≥5) were shown above. Here, we try to show the robustness of the patterns by using the high-confidence mutations with much stricter cutoffs (coverage ≥20, alternative allele ≥3, and also absent in the 1000-genomes SNPs. See Methods for details).

**Fig. 7:**
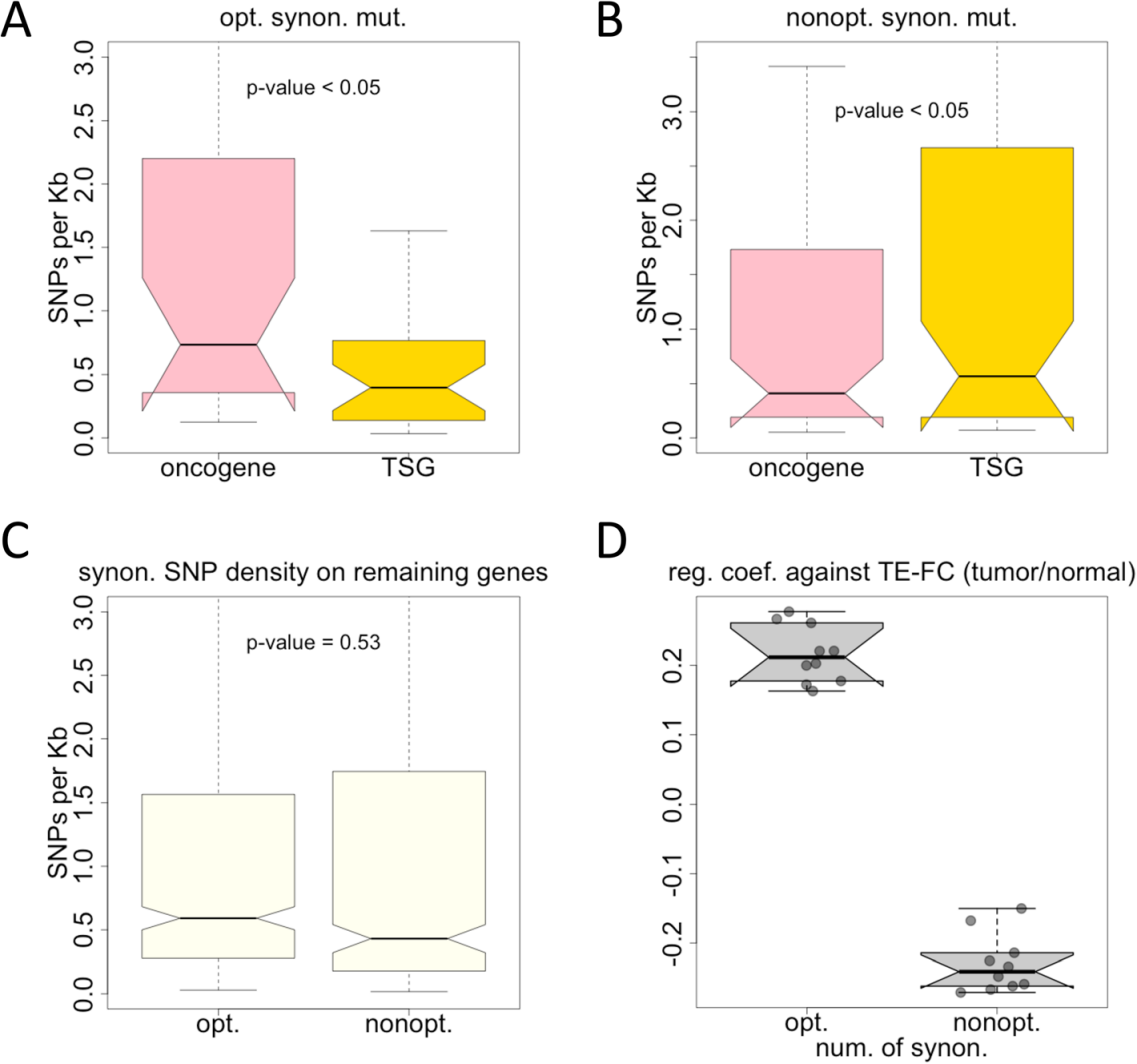
SNP density (number of SNPs per Kb CDS) in different genes. The high-confidence SNPs were used in this part (coverage ≥20, alternative allele ≥3, and absent in 1000-genomes). **a** Optimal synonymous SNP density in oncogenes and TSG. KS test was used to calculate p-value. **b** Non-optimal synonymous SNP density in oncogenes and TSG. KS test was used to calculate p-value. **c** Synonymous SNP density in the remaining genes. KS test was used to calculate p-value. **d** Regression coefficients of “Y ~ X1 + X2 + ...Xn”. Y is the TE fold change of tumor samples to normal samples. The variables included the number of optimal synonymous mutations and the number of non-optimal synonymous mutations

We validated the following patterns. The density of optimal synonymous mutations is significantly higher in oncogenes than in TSG (Fig. 7a), and the density of non-optimal synonymous mutations is significantly higher in TSG than in oncogenes (Fig. 7b). No significant difference of SNP densities (optimal versus non-optimal) was observed on the remaining genes (Fig. 7c). The optimal synonymous mutations contribute positively to the gene TE while the non-optimal synonymous mutations negatively affect the gene TE (Fig. 7D). All these patterns suggest that our observation of synonymous mutations and codon optimality affecting the translation elongation speed is robust.

## Discussion

Intuitively, the oncogenesis process might be caused by the enhancement of oncogenes or the suppression of TSG. The enhancement or suppression could be regulated at (1) transcription level; (2) mRNA decay level; (3) translation level; (4) protein degradation level; or (5) protein activity level. In this study, we focused on the alteration at translation level, and has associated this translational change with the synonymous mutations in tumor samples. This is a novel angle that interprets the cancer development with synonymous codon usage bias. Our study hints that the synonymous mutations could affect the codon optimality and consequently regulate the translation of codons. The impact of synonymous mutations is detectable, and it might more or less contribute to the cancer development.

In early years, the literatures on synonymous codon usage bias mainly analyzed its evolutionary patterns based on genome sequences. Recently, as many technologies improved, the effect of synonymous codon usage bias could be demonstrated by artificially manipulating the codon sequence [55]. However, we claim that the experimental manipulation of codon sequences has several concerns that should consider: (1) The control group (wildtype sequence) and experimental group (altered sequence) might differ in trans factors, and thus the sequence alone might not be able to completely explain the different behavior. But the heterozygotes or hybrid systems well solve this problem. (2) The patterns found in lower organisms (like bacteria and yeasts) might not be suitable for humans. (3) Most literatures studied the effect of codon usage bias on transcription level, mRNA decay level, and protein folding level. No evidence of translational changes has been observed in vivo or in vitro.

Therefore, our study nicely filled these gaps. (1) The use of human tumor samples avoided the concern and uncertainty of using other animals. (2) The focus on heterozygous SNPs fully took advantage of the identical trans environment. The different behavior observed on the two alleles could only be explained by sequence discrepancies. (3) Translational regulation bridged the gap between mRNA and protein.

One may ask that in this study the translational regulation exerted by synonymous mutations was examined at nucleotide level or codon level, but why this regulation could not be directly tested at gene level by using the ribosome profiling data? One should be clear that the translation initiation process and translation elongation speed work together to determine the protein production rate. However, translation initiation is the rate limiting step and serves as the major determinant of protein production. Thus, the term translation efficiency (TE) usually specifically refers to the translation initiation efficiency, while elongation speed only fine-tunes the amount of proteins. In the ribosome profiling data, the RPF reads count per gene divided by the RNA reads count per gene could roughly reflect the translation initiation efficiency of a gene. This algorithm automatically assumes that the ribosome moves at constant speed on the mRNA. Therefore, the effect of synonymous mutations on protein production rate is very minor and is not likely to be detected at gene level by ribosome profiling data.

However, since the ribosome profiling data has the ability to capture the codon-resolution (or nucleotide-resolution) translation events, if one only used these data to calculate the gene-level TE, then it did not fully utilize the information hidden in the data. We understand the advantage of ribosome profiling and we have done what we could to parse the data at nucleotide-resolution, willing to reflect the differential elongation speed on the two alleles. We did so. Nevertheless, the alteration in elongation speed is not impactful enough to be detected at gene level. The ribosome profiling data alone could not directly testify the final protein abundance of the two alleles. All in all, we believe that our results we provided were adequate to support our conclusion that the synonymous mutations could regulate the translation elongation speed.

## Conclusions

Synonymous mutations might play a role in liver cancer development by altering codon optimality and translational velocity. Synonymous mutations should no longer be ignored in the genome-wide studies.

## Data Availability

The human and macaque reference genomes were downloaded from Ensembl (http://ensemblgenomes.org/). The sequencing data (RNA-seq and ribosome profiling) of normal/tumor tissues from ten patients were downloaded from NCBI via accession number GSE112705. The lists of oncogenes and TSG were downloaded from cancer gene consortium (CGC, https://cancer.sanger.ac.uk/census/). The SNP data of the 1000 genome project were downloaded from official website (ftp://ftp.1000genomes.ebi.ac.uk/). The genome version is “phase2 reference assembly sequence hs37d5”.

## Abbreviations

SNP: single nucleotide polymorphism
CDS: coding sequence
UTR: untranslated gene
RPF: ribosome protected fragments
TE: translation efficiency
TSG: tumor suppressor gene
dN: nonsynonymous substitution rate
CGC: cancer gene consortium
tRNA: transfer RNA
